# Malignant transformation of oral lichen planus: where are we now?

**DOI:** 10.4317/medoral.26834

**Published:** 2024-10-13

**Authors:** Miguel Ángel González-Moles, Pablo Ramos-García

**Affiliations:** 1 School of Dentistry, University of Granada, Granada, Spain; 2 Instituto de Investigación Biosanitaria ibs.GRANADA, Granada, Spain

## Abstract

**Background:**

Oral lichen planus (OLP) is a very prevalent disease whose main clinical feature is the appearance of white hyperkeratotic reticular lesions, which may or may not be accompanied by erosive and/or atrophic lesions, among others. One of the most relevant aspects of the process is its current consideration as an oral potentially malignant disorder (OPMD), although this is currently the subject of considerable controversy.

**Material and Methods:**

A review of the literature was carried out in order to critically analyze the controversies surrounding the consideration of OLP as an OPMD, where they originate from and the available evidence that has led to the conclusion that OLP patients are at risk of developing oral cancer.

**Results:**

The controversies over the definitive acceptance of OLP as an OPMD were classified as controversies related to the lack of widely accepted diagnostic criteria for OLP; controversies related to histopathological aspects of OLP and the presence of epithelial dysplasia as a diagnostic exclusion criterion; and controversies related to clinical aspects of OLP (which in turn were subclassified into: controversies on how to interpret reticular lesions in OLP, on the nature of the white plaques that appear in OLP; on the changing character of reticular lesions in OLP; and on the criteria for accepting a case as a true malignant OLP). Furthermore, evidence to justify the acceptance of OLP as an OPMD was in depth reviewed, including the molecular evidence, evidence from research studies with the highest evidence design -systematic reviews and meta-analyses-, and evidence from case series reporting strong results.

**Conclusions:**

This paper presents the reasons for the controversies as well as the evidence that allows us to accept that OLP behaves as an OPMD.

** Key words:**Oral lichen planus, malignant transformation, oral potentially malignant disorder, cancer.

## Introduction

Lichen planus is an autoimmune disease (1) with priority involvement of the oral mucosa (OLP) that can also affect the skin, scalp, nails, as well as other mucous membranes (2). It is a common disease (3) affecting approximately 1% of the general population, with geographical differences in prevalence, Europe being the most prevalent in the world (1.36% of the general population); it also occurs significantly more frequently after the age of 40 years (3). The determining clinical fact in OLP is the appearance of hyperkeratotic white reticulae located in different areas of the oral mucosa, which may or may not be accompanied by atrophic, erosive, plaque-like or, more infrequently, bullous lesions (2). In 2020, a meeting of experts on oral potentially malignant disorders (OPMD) was held in Glasgow, convened by the WHO Collaborating Centre for Cancer Research, which considered OLP as a disease at risk of progressing to oral cancer (4), although it must be acknowledged that there is still considerable controversy about this fact.

This paper presents the controversies surrounding the consideration of OLP as an OPMD, where they originate from and the available evidence that has led to the conclusion that patients with OLP are at risk of developing oral cancer.

## Material and Methods

Literature was searched in this narrative review in order to carefully and critically analyze the precedent objectives. MEDLINE was considered as the main electronic database and searched -through PubMed- for studies published before Jun-2024 (upper limit), without lower date limit. Multiple searches were conducted by combining thesaurus MeSH terms with free terms, constructed to maximize sensitivity.

In a first line general search, the root keyword was “oral lichen planus”, which in terms of sensitivity provides a very broad search strategy. In addition, several more precise searches were subsequently conducted by combining relevant aspects of the subsections to be reviewed (i.e., the controversies and evidences surrounding the consideration of OLP as an OPMD). We also manually screened the reference lists of retrieved studies for additional relevant studies. Most of the studies were included or excluded according to an exhaustive analysis of the title, abstract, year of publication, impact factor of the journal and number of citations received. Although these last two criteria may introduce a potential selection bias, its application is necessary when handling several large number of records across multiple search strategy rounds, e.g., ("Lichen Planus, Oral"[mh] OR "oral lichen planus"[all] OR "olp"[tiab] OR "oral lichenoid lesion"[all] OR "oll"[tiab]) AND ((“potentially”[all] AND “malignant”[all] AND disorder*[all]) OR “Mouth Neoplasms”[mh] OR malign*[all] OR premalign*[all] OR "Carcinoma, Squamous Cell"[mh] OR "oscc"[tiab] OR “transformation”[tiab] OR "risk"[tiab] OR "progression"[ tiab]).

## Results

The controversies over the definitive acceptance of OLP as an OPMD were classified as controversies related to the lack of widely accepted diagnostic criteria for OLP; controversies related to histopathological aspects of OLP and the presence of epithelial dysplasia as a diagnostic exclusion criterion; and controversies related to clinical aspects of OLP (which in turn were subclassified into: controversies on how to interpret reticular lesions in OLP, on the nature of the white plaques that appear in OLP; on the changing character of reticular lesions in OLP; and on the criteria for accepting a case as a true malignant OLP).

Furthermore, evidences to justify the acceptance of OLP as an OPMD were in depth reviewed and classified into molecular evidence, evidence from research studies with the highest evidence design -systematic reviews and meta-analyses-, and evidence from case series reporting strong results.

## Discussion

- Controversies over the definitive acceptance of OLP as an OPMD

**Figure 1:**
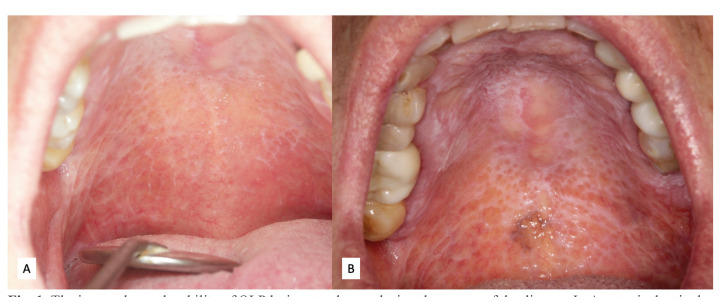
The image shows the ability of OLP lesions to change during the course of the disease. In A, a typical reticular appearance is observed, which after the evolution of the lesion over several years (B) develops areas of verrucous plaque-like aspect and areas of hyperpigmentation.

OLP is in some ways an enigmatic disease due to the multiple clinical forms in which it can present, the subtlety with which its most typical lesions occasionally manifest themselves, the changing nature of its clinical manifestations and also its current consideration as an OPMD (Fig. 1).

In 2008, some members of our research group, in collaboration with Professor Crispian Scully, published a first paper analysing the main controversies and difficulties in the consideration of OLP as an OPMD (5), and we must recognise that many of these controversies persist today.

The controversies that have been mentioned could be categorised as follows:

1. Controversies related to the lack of widely accepted diagnostic criteria for OLP.

The very complexity of the clinical presentation of OLP and its changing appearance makes it difficult to establish precise criteria for the diagnosis of the disease (2). Ideally, diagnostic criteria for OLP should be proposed by expert groups, consensual and evidence-based. A first consideration concerns what is considered to be an ‘expert in OLP’. In our opinion, this should be a clinician and researcher who has proven experience in the diagnosis and management of patients with OLP in all its dimensions, and who also has a proven trajectory in OLP research with results published in JCR journals. The proposed criteria should be consensus-driven and evidence-based, which implies that they should only be derived from the most robust knowledge, i.e. large case series and well-constructed systematic reviews and meta-analyses published in relevant journals in the field. As will be seen below, all of the diagnostic criteria published to date fail to me*et al*l of these requirements, either because they are derived from proposals by very small groups of authors -two or three authors- or because they have been proposed by clinicians or researchers who do not meet the requirement of being experts in OLP, and finally, and above all, because they are not evidence-based.

The first germ for diagnostic criteria for OLP appeared in 1978 in the journal Oral Surgery, Oral Medicine, and Oral Pathology and derived from a group of eminent scientists and clinicians of that time who belonged to a WHO-elected working group led by I.R. Kramer (6). That paper was really a description of the disease in which a series of clinical and histopathological facts that the authors considered relevant were presented (Table 1) and not a formal proposal of diagnostic criteria; however, in our opinion, two facts from that paper deserve to be highlighted: first, the authors state that cases of malignant OLP had previously been published, although the frequency of the transformation phenomenon was unknown and, second, nothing was reported in that paper about the presence of epithelial dysplasia in OLP, which, as we will see below, constitutes one of the axial aspects of the controversy.

Subsequently, in 2003, van der Meij and van der Waal (7), studying the degree of agreement among clinicians in the diagnosis of OLP using the recommendations issued by the WHO in 1978, realising that this was low and hoping to improve it, proposed a set of criteria (Table 2) that considered clinical and histopathological aspects, with the presence of bilateral reticular symmetrical lesions and the absence of epithelial dysplasia being essential for the diagnosis of OLP. All lesions that did not fulfil these strict criteria should, at the authors' suggestion (7), be referred to as oral lichenoid lesions (OLL). However, the authors themselves acknowledged in their paper that (literally) ‘We realise that the application of these criteria will exclude a number of patients who may actually have the disease but who do not meet the strict criteria’. These criteria have generally been called in the literature as ‘modified WHO criteria’ although, as can be deduced from reading the paper, the WHO has in no case expressed acceptance of these criteria as their own. We must recognise that the criteria proposed by van der Waal have had an extraordinary impact among clinicians and researchers who have accepted them for many years without any questioning; however, as we will see below, their main drawback is that they are not based on evidence.

Later, in 2016, the American Academy of Oral and Maxillofacial Pathology published a position paper on OLP with proposed diagnostic criteria (Table 3) that considered clinical and pathological aspects (8), including the presence of multifocal and symmetrical lesions, and the absence of epithelial dysplasia and verruciform architecture in the affected epithelium, among others. As can be noted, these criteria would allow the diagnosis of a lesion as OLP without the presence of reticular lesions; moreover, as will be seen below, these criteria were not evidence-based either.

Finally, in 2020, the authors of this paper published diagnostic criteria for OLP (9) (Table 4) in which, for reasons to be discussed, bilaterality and symmetry of lesions are not considered essential, nor is the presence of dysplasia accepted as an exclusion criterion for the diagnosis of OLP.

2. Controversies related to histopathological aspects of OLP. The presence of epithelial dysplasia as a diagnostic exclusion criterion.

Some of the diagnostic criteria for OLP (7,8) consider the presence of epithelial dysplasia as an exclusion criterion. We have previously published that this approach results in an underestimation of the risk of the malignancy of the disease (10). Obviously, if primary level studies focusing on OLP malignancy are designed to exclude cases with epithelial dysplasia, since dysplasia is the most important determinant of cancer risk in OPMD, the consequence will be a reduction in the reported malignancy rate; this has been confirmed in a recent meta-analysis (11) of 12838 cases of OLP of which 151 (1.2%) malignant, however, when the authors apply strict criteria for the diagnosis of OLP -essentially using the presence of dysplasia as an exclusion criterion- the malignancy rate falls to 0.44%.

**Figure 2:**
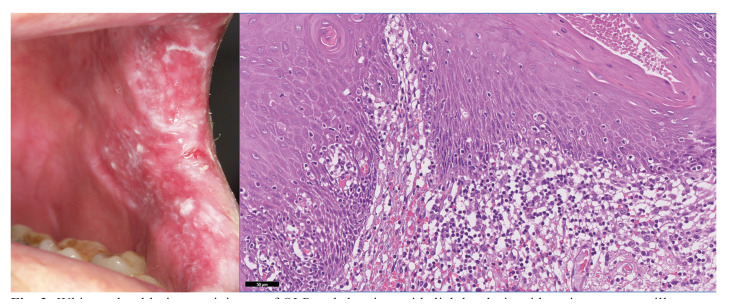
White and red lesion reminiscent of OLP and showing epithelial dysplasia with an immunosurveillance response with histopathological findings of lichenoid appearance. This case should not be considered as a true malignant OLP according to Krutchkoff.

The origin of the controversy can be found in the publication of David Krutchkoff (12) in 1978, who, reviewing the cases of malignant OLP published up to that date, observed that most of them corresponded to white and red lesions that could resemble OLP and that developed epithelial dysplasia in which an immunosurveillance response was established with lichenoid facts (Fig. 2), and therefore, only a minority (15 out of 233 published cases) would, according to the author, be true malignant OLP; For Krutchkoff this fact undeservedly overestimated the real capacity of OLP to progress to oral cancer and for this reason he presented exclusively histopathological criteria for the diagnosis of OLP, being the first to propose that OLP did not develop epithelial dysplasia (13). This paper is probably acknowledging that the assessment of epithelial dysplasia in OLP is complex, and on this most authors agree (14), although this fact does not exclude in our opinion that OLP may develop dysplasia on the way to malignant transformation as do the other OPMDs. It should be noted that Krutchkoff in his paper does not offer any evidence-based reason to justify the proposed criterion, despite which it was accepted by the scientific community without dispute for many years (12,13). However, a thorough review of the scientific literature on the subject yields information to the contrary, i.e. OLP can develop epithelial dysplasia and this has a determining impact on their risk of malignancy. The evidence for this assertion comes from reported case series of OLP, the most relevant of which, in the opinion of the authors of this paper, are selected and summarised below. In 2023, a group of eminent clinicians and researchers from the Eastman Dental Institute and other institutions, led by Pimolbutr (15), analysed the parameters affecting the prognosis of epithelial dysplasia in patients with and without OLP. The authors, based on the study of 299 cases of oral epithelial dysplasia whose samples were in the archives of their histopathology laboratory, proceeded to review the patients' records. Some of the results are relevant: 144 of 299 patients presenting with epithelial dysplasia (48.16%) had previously been diagnosed with OLP following criteria that included bilaterality of the lesions and absence of dysplasia, indicating that the development of dysplasia in previously non-dysplastic OLP was not only possible but frequently appeared in the series; Patients with OLP who developed oral epithelial dysplasia were also significantly predisposed to the appearance of new areas of dysplasia in oral locations other than the initial one; which catalogues OLP as a risk factor for the development of multiple areas of dysplasia in the oral mucosa; and finally, the malignancy rate of dysplasia preceded by OLP (45%) was not significantly different from the malignancy rate of dysplasia in patients without OLP (54%) (*p*=0.68), from which it could be deduced that OLP essentially becomes malignant through the development of dysplasia and that once dysplasia is established, it becomes the most relevant driver of malignant transformation. In another paper in 2018 (16), a relevant group of authors from the University of British Columbia, studying lichenoid mucositis -in which they integrate cases of OLP and cases of lichenoid drug reactions- conclude that lichenoid mucositis with dysplasia should be considered premalignant, that lichen planus is more likely to develop dysplasia than normal mucosa, and that clinicians should not ignore dysplasia in the diagnosis of pathology, even if they believe the patient has lichen planus clinically. In 2024, the journal Oral Diseases published a paper (17) on the facts of autoimmunity and epithelial dysplasia in patients with oral lichenoid disease, which was composed of OLP cases, OLL cases and cases of lichenoid drug reactions. This paper reports that among the 66 patients in the OLP group, 14 cases (21%) developed dysplasia, which was significantly more frequent in patients with a previous history of cancer in any location of the organism, in cases of erosive OLP and in those affecting the tongue.

The strongest evidence to accept that OLP can develop dysplasia on the path to malignancy comes from two systematic reviews and meta-analyses that have compared malignancy rates of OLP with dysplasia vs. OLP without dysplasia (18,19) and reported that cancer in OLP with dysplasia develops in 6.22% and 5.13% vs. 1.14% and 1.43% of malignant cases of OLP without dysplasia, respectively, these comparisons being statistically significant.

**Figure 3:**
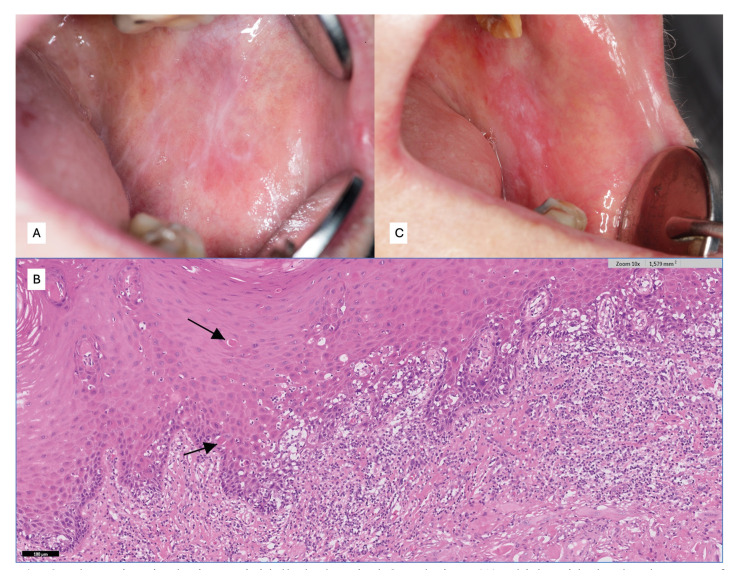
The patient in the image initially had typical OLP lesions (A) which, with the development of epithelial dysplasia, with lichenoid features (black arrows), acquired a clinical appearance without lichenoid features (C).

Finally, in a position paper of our research group on the relevance, controversies and challenges of the evaluation of oral epithelial dysplasia in OLP (14) we concluded, after a thorough review of the literature on the subject, that there are no evidence-based reasons to consider epithelial dysplasia as a diagnostic exclusion criterion for OLP and that it should therefore be evaluated by experienced pathologists taking into account the clinical aspects of the lesion at the time of biopsy, but also, and especially, the initial clinical aspects of the lesion before the development of epithelial dysplasia. This last consideration is relevant because one might ask, in the opinion of the author of this paper, what remains of typical OLP lesions, both clinically and histopathologically, once epithelial dysplasia is established and especially progresses to higher degrees of severity? In the author's personal experience, the clinical appearance of the typical OLP lesion may change with the establishment and progression of epithelial dysplasia to such an extent as to make the case unrecognisable as an OLP unless the clinician has prior knowledge of the patient's history and evolution (Fig. 3), and this probably also occurs in the histopathological sphere as seems to be demonstrated in this paper (20) in which the severity of dysplasia was associated with a lower frequency of lichenoid histological findings.

These reasons, in conclusion, lead us to consider that dysplasia can be present in OLP and that it should not be accepted as a diagnostic exclusion criterion as we reflect in our proposed diagnostic criteria for OLP.

3. Controversies related to clinical aspects of OLP:

On how to interpret reticular lesions in OLP.

**Figure 4:**
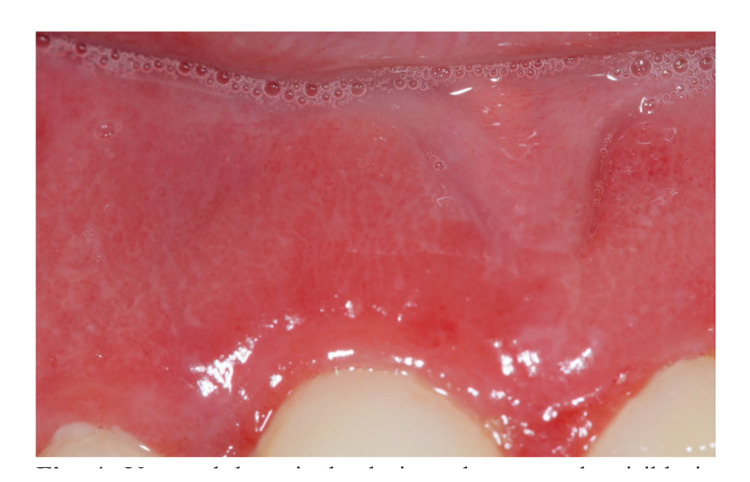
Very subtle reticular lesions that are only visible in high-magnification photographs.

If there has been almost general agreement in relation to OLP, it has come from the consideration of reticular lesions as a determining fact in the diagnosis of the disease. However, sometimes the reticular appearance is so subtle that it may occasionally go unnoticed, and thus, in the author's personal experience, the presence of white reticules is sometimes only seen when viewing good quality pictures at high magnification (Fig. 4).

Thus, for some clinicians these lesions will go unnoticed and in other cases they will not be sufficiently accepTable to consider a case with these features as a true OLP. This is not a trivial issue because if a clinical case with these subtle lesions eventually develops into an oral carcinoma, it may or may not be considered a malignant OLP depending on whether the clinician has considered its reticules accepTable for the diagnosis of the disease.

On the consideration of bilaterality and symmetry of lesions as essential criteria for the diagnosis of OLP.

Some proposals for diagnostic criteria for OLP (6-8) with wide repercussion in the scientific literature have considered bilaterality and symmetry of lesions as essential facts for diagnosis and, again, many clinicians and researchers have accepted this proposal without any questioning. This idea was first put forward by van der Meij and van der Waal in 2003 (7). Subsequently, the same authors (21) studied the malignancy rate of their series of 192 cases of OLP and OLL diagnosed according to their own criteria and reported that only four cases progressed to oral cancer, all of them OLLs, i.e. unilateral or frankly asymmetric lesions; for this reason they considered OLP as a completely benign lesion that did not deserve special procedures aimed at early diagnosis of oral cancer, i.e. applying follow-up protocols. Again, this consideration was accepted by many clinicians and researchers. However, the scientific evidence on the subject does not allow us to accept that OLP is a lesion without risk of developing cancer, nor that the risk of malignancy falls exclusively on OLLs. The only three systematic reviews and meta-analyses comparing the malignancy rate of OLP vs. OLLs show that OLP can progress to cancer at a rate not different from that of OLLs (9,18,19). Consequently, OLLs are not the only ones that become malignant, nor do they significantly more often become malignant than OLP, and therefore, there is no justification for categorising bilateral lesions as OLP and as a different entity from unilateral or frankly asymmetric lesions (OLL), and this is reflected in our diagnostic criteria: we do not consider bilaterality or symmetry of the lesions as essential facts for the diagnosis of OLP (9).

Careful reflection on this change in nomenclature regarding OLP, in particular on the relevance of creating a subgroup of OLP-like lesions called OLL, has led us to the consideration that this change in nomenclature is not only unjustified but potentially very detrimental to patients. In 2017, Doust *et al* (22) published in JAMA Internal Medicine a checklist to guide the modification of disease definitions, specifically in relation to when it would be indicated to modify the definition of a previously known disease or when a disease should be separated from another previously defined disease. The authors proposed a set of criteria that should be considered before assigning a new nomenclature. Among these criteria the following stand out in our view: The authors wonder how the new definition would change the prevalence of the preceding disease; in our opinion, acceptance of the existence of OLLs -in the sense of van der Waal's proposal (7)- would markedly and arbitrarily decrease the prevalence of OLP in favour of OLLs. A second criterion refers to whether the trigger for the new definition is justified; as we have discussed, the justification for the change would be that OLP does not become malignant and OLLs do, and since this is not supported by the evidence, consequently the change in nomenclature is not justified. And finally, what would be the benefits and harm that the new definition could generate for patients; in our opinion, the harms are very relevant because the unjustified consideration of OLP as a non-risk lesion would eliminate the necessary care that patients require regarding the early diagnosis of cancer -lifelong follow-up of patients- and would dangerously modify the information that should be transmitted to patients about their process, i.e. patients should be informed that they have an OPMD.

On the nature of the white plaques that appear in OLP.

Among the clinical variants that can occur in OLP are white hyperkeratotic plaques, which form the so-called plaque type lichen planus variant. Consideration of the nature of these plaques is determinant in the assessment of the prognosis of patients and in the analysis of the malignancy rates of the disease. In relation to this aspect, several situations could arise: first, some plaque lesions stand out above the reticular and atrophic lesions, which in some cases could be very subtle; some clinicians and researchers will consider these lesions as leukoplakia and thus, if a carcinoma eventually develops, this would be considered as a case of malignant oral leukoplakia. On this aspect there is no agreement, and even a debate has not been raised. Second, some white plaques in the context of OLP lesions present histology without lichenoid facts; in such a case the question is what is the relationship between the two types of lesions -reticules with lichenoid facts and white plaques without lichenoid facts-. Third, some patients with erosive and atrophic forms of OLP develop white plaques after treatment of their lichen planus with corticosteroids; the question would then be what relevance these plaques have to malignant processes, this aspect is unresolved.

On the changing character of reticular lesions in OLP.

Clinical experience, especially based on long-term follow-up of patients with OLP, has shown that in some patients the initial lesions typical of OLP evolve into more heterogeneous forms of the disease, essentially with the development of white plaques with some verrucous areas extending over large areas of the oral mucosa, with or without retaining some of the initial appearance of OLP. These lesions would correspond to the so-called cases of OLP that evolve into proliferative verrucous leukoplakia (PVL), and there are some case series that describe the particularities of these lesions (23,24). However, there are still some unanswered questions about this process for which there is insufficient scientific evidence; some of them could be the following: first, do these lesions that evolve to PVL behave clinically as true PVL in the sense that Hansen initially described them in 1984 (25)? Second, do these lesions have the same potential to progress to cancer as PVL (26,27)? Third, in case of cancer development, which should be considered premalignant, OLP, or both?

On the criteria for accepting a case as a true malignant OLP.

The first criteria for accepting a case as a true malignant OLP were proposed by Krutchkoff *et al* (12) in 1978. For the author, all reported cases of cancer in OLP should have been diagnosed at least two years after the diagnosis of the OLP, although no explanation is offered to justify the proposal. This criterion has been followed by some authors, although the time intervals between the development of cancer and the initial diagnosis of OLP have been modified -6 month - without explanation for the change (11). The justification for this criterion could be found in the possibility that the oral mucosa may develop a lichenoid appearance (reticulation, atrophy and/or erosions) as a phenotypic response to the immunosurveillance phenomenon developed against the cancer. This, although possible, does not exclude that some patients may present to the office with concomitant OLP and cancer, especially if we take into consideration that OLP is often underdiagnosed and that patients may have an undiagnosed disease for a long time and only come to the oral medicine office when they perceive a worrisome lesion. The final decision on whether to accept a case of concomitant presentation of OLP and cancer as a true malignant OLP will only depend on whether an experienced clinician can deduce from the clinical history information that the OLP lesions preceded the neoplasm, and on clinical examination finds OLP lesions spread over wide areas of the oral mucosa and not only localised to the area of the cancer.

Krutchkoff *et al* (12) also proposed as a restriction that acceptance of a case as a true malignant OLP would require the cancer to appear in an area affected by OLP lesions. This is, in the experience of the author of this paper, frequently observed in malignant OLP. However, numerous authors have pointed out that patients with OPMD are at risk of developing oral cancer at any location of the oral mucosa, even with a normal clinical appearance (4); moreover, an interesting paper has reported that 36% of upper aerodigestive tract mucosa of normal clinical appearance accompanying head and neck carcinomas have molecular alterations predisposing to cancer (loss of heterozygosity on 3p, 9p and 17p) (28).

Finally, Krutchkoff *et al* (12) have also considered that only those occurring in non-smokers could be accepted as true malignant OLP, blaming the cancer on the effects of smoking in cases of malignant OLP in smokers. Some authors have disagreed with this criterion (29) as it does not take into account the possibility of cooperation of two different factors that could raise the risk to levels higher than the simple additive effect (5). Moreover, evidence shows that OLPs in non-smokers also become malignant (18,19).

- Evidence to justify the acceptance of OLP as an OPMD

1. Molecular evidence

In recent years, our group has developed a line of research analysing the expression of cell cycle and apoptosis regulatory proteins in the OLP (30-35). The results of this line on case series report that the OLP epithelium frequently develops a proliferative and anti-apoptotic activity with frequent expression of p53 protein that presumably occurs at the expense of its normo-functioning wild type. We have interpreted this frequent pattern of response as a mechanism aimed at maintaining the integrity of the oral mucosal epithelium by preserving epithelial cells with the development of anti-apoptotic (Bcl-2 overexpression, caspase 3 downregulation), hyperproliferative (substance *P* and Ki-67 overexpression) and genome-protective (p53 overexpression) stimuli. However, we also know that the oral epithelium affected by lichen planus is subject to oncogenic effects linked to the inflammatory infiltrate itself (iNOX, COX-2, various interleukins, etc.); therefore, the diseased epithelium, by developing these survival mechanisms linked to hyperproliferation and resistance to apoptosis, is probably also exposed to a risk of malignisation if the genome's protective mechanisms fail. Furthermore, in this line of research we have also studied on the basis of evidence to what extent proteins that are considered to be hallmarks of cancer cells are expressed in oral epithelium affected by lichen planus (34,35) and found a very common expression of these cancer markers in the OLP epithelium.

Further evidence comes from the demonstrated likelihood that some autoimmune diseases (Hashimoto's thyroiditis, ulcerative colitis, Sjögren's syndrome, etc.) are likely to develop cancer in the affected organ, by a mechanism linked to chronic autoimmune inflammatory aggression (36); in our opinion, logic dictates that if this occurs in some autoimmune diseases, it is also accepTable that it can occur in oral lichen planus.

2. Evidence from research studies with the highest evidence design - systematic reviews and meta-analyses.

To date, seven systematic reviews and meta-analyses have been published on the malignancy of OLP (9,11,18,19,37-39) and all of them offer very similar malignancy rates; the one offering the lowest rate (0.44% of cases) (11) applies very strict criteria, already discussed above, for the diagnosis of OLP and for accepting a case as a malignant OLP; the paper offering the highest malignancy rate (2.28%) comes from our research group (9) and meta-analyses the top 10 papers selected for their best methodological quality, which seems to indicate that as research is better executed, more cases of malignancy are reported.

In addition, a systematic review and meta-analysis (40) shows that oral carcinomas developed on OLP have different prognostic behaviours compared to conventional carcinomas, especially with regard to their lower 5-year mortality ate (15% of cases) which is significantly lower than that usually reported for conventional carcinoma (50% of cases). Evidence has also shown that OLP behaves like a cancerisation field, and thus, when a patient affected by OLP develops a first carcinoma, he/she has an 11% chance of developing new cancers (40).

3. Evidence from case series reporting strong results.

In this section, specific mention should be made of the work of Reeve *et al* (1) who analyse data from the Finn Gen study, a project that integrates genome information from a cohort of 473,000 Finns with data from their medical records. The authors find that in that overall cohort, 3323 patients had a history of OLP. The conclusions of this prestigious study are compelling: patients with OLP have a significantly increased risk for the development of oral cancer in general (OR=9.6) but most uniquely for the development of tongue cancer (OR=13.6); furthermore, the malignant transformation rate of OLP in Finland was 1.9% over 10 years (63/3323), which compared to the 0.2% of oral cancer in the whole cohort, confirms a strong risk of oral cancer for OLP.

## Conclusions

The results derived from the most evidence-based studies allow attributing a premalignant character to OLP and thus, this disease should be considered as an OPMD. This fact is relevant because it makes it necessary to clearly inform patients about their cancer risks and to establish follow-up protocols, probably for life. Authors who doubt the potentially malignant nature of OLP generally base their arguments on facts that are not supported by the evidence (especially considering epithelial dysplasia as a diagnostic exclusion criterion for the disease and accepting that OLP maligns and OLP does not) or on proposals of arbitrary criteria that are not justified, essentially those designed to accept malignant OLP cases as genuine. There is also a nomenclature problem regarding lesions presenting with erosive reticular and/or atrophic appearance (OLP, OLL, lichenoid reactions, lichenoid mucositis, etc.), which is still unresolved and continues to generate debate. Our recommendation is that all reticular and atrophic/erosive lesions should be considered at risk for progression to cancer regardless of their name.

## Figures and Tables

**Table 1:** Diagnostic criteria for OLP (Kramer, Lucas, Pindborg and Sobin 1978).

Diagnostic criteria	
Clinical features	- Mucosal lesions are usually multiple and often have a symmetrical distribution.
	- They commonly take the form of minute white papules which gradually enlarge and coalesce to form either a reticular, annular, or plaque pattern.
	- A characteristic feature is the presence of slender white lines (Wickham's striae) radiating from the papules. In the reticular form there is a lacelike network of slightly raised gray-white lines, often interspersed with papules or rings.
	- The plaque form may be difficult to distinguish from leukoplakia, but in lichen planus there is usually no change in the flexibility of the affected mucosa.
	- In some patients the lesions are atrophic, with or without erosions.
	- Oral lesions of lichen planus may also include bullae, but these are rare.
Histopathological features	- There is usually a keratinized layer, and this may be either ortho- or parakeratinized.
	- The "saw tooth" appearance of the rete processes that is a common feature of skin lesions is less frequently seen in the oral mucosa.
	- The thickness of the epithelium varies, and atrophy is often seen.
	- Civatte (colloid) bodies may be present in the region of the basal-cell layer, lying either in the epithelium or within the superficial part of the connective tissue. These are rounded or lobulated acidophilic structures which sometimes contain a pyknotic nucleus or nuclear fragments.
	- The changes in the basal-cell layer often include liquefaction degeneration, and there may be a narrow band of eosinophilic material in the position of the basement membrane.
	- There is a well-defined zone of cellular infiltration that is confined to the superficial part of the connective tissue (lamina propria), and the infiltrate consists mainly of lymphocytes except in the vicinity of an erosion.
	Source: Kramer, I. R., Lucas, R. B., Pindborg, J. J., & Sobin, L. H. (1978). Definition of leukoplakia and related lesions: an aid to studies on oral precancer. Oral surgery, oral medicine, and oral pathology, 46(4), 518-539.

**Table 2:** Diagnostic criteria for OLP (van der Meij and van der Waal 2003).

Diagnostic criteria	
Clinical criteria	- Presence of bilateral, more or less symmetrical lesions
	- Presence of a lace-like network of slightly raised gray-white lines (reticular pattern)
	- Erosive, atrophic, bulbous and plaque-type lesions are only accepted as a subtype in the presence of reticular lesions elsewhere in the oral mucosa.
	- In all other lesions that resemble OLP but do not complete the aforementioned criteria, the term `clinically compatible with´ should be used.
Histopathologic criteria	- Presence of a well-defined band-like zone of cellular infiltration that is confined to the superficial part of the connective tissue, consisting mainly of lymphocytes
	- Signs of `liquefaction degeneration' in the basal cell layer
	- Absence of epithelial dysplasia
	- When the histopathologic features are less obvious, the term `histopathologically compatible with´ should be used
Final diagnosis OLP or OLL	To achieve a final diagnosis clinical as well as histopathologic criteria should be includedOLP - A diagnosis of OLP requires fulfillment of both clinical and histopathologic criteria OLL - The term OLL will be used under the following conditions:
	1. Clinically typical of OLP but histopathologically only `compatible with´ OLP
	2. Histopathologically typical of OLP but clinically only `compatible with´ OLP
	3. Clinically `compatible with´ OLP and histopathologically `compatible with´ OLP
	Source: van der Meij, E. H., & van der Waal, I. (2003). Lack of clinicopathologic correlation in the diagnosis of oral lichen planus based on the presently available diagnostic criteria and suggestions for modifications. Journal of oral pathology & medicine : official publication of the International Association of Oral Pathologists and the American Academy of Oral Pathology, 32(9), 507-512.

**Table 3:** Diagnostic criteria for OLP (Cheng, Gould, Kurago, Fantasia and Muller 2016).

Diagnostic criteria	
Clinical criteria	- Multifocal symmetric distribution
	- White and red lesions exhibiting one or more of the following forms:
	-- Reticular/papular
	-- Atrophic (erythematous)
	-- Erosive (ulcerative)
	-- Plaque
	-- Bullous
	- Lesions are not localized exclusively to the sites of smokeless tobacco placement
	- Lesions are not localized exclusively adjacent to and in contact with dental restorations
	- Lesion onset does not correlate with the start of a medication
	- Lesion onset does not correlate with the use of cinnamon-containing products
Histopathologic criteria	- Band-like or patchy, predominately lymphocytic infiltrate in the lamina propria confined to the epithelium- lamina propria interface
	- Basal cell liquefactive (hydropic) degeneration
	- Lymphocytic exocytosis
	- Absence of epithelial dysplasia
	- Absence of verrucous epithelial architectural change
	Source: Cheng, Y. S., Gould, A., Kurago, Z., Fantasia, J., & Muller, S. (2016). Diagnosis of oral lichen planus: a position paper of the American Academy of Oral and Maxillofacial Pathology. Oral surgery, oral medicine, oral pathology and oral radiology, 122(3), 332-354.

**Table 4:** Diagnostic criteria for OLP (González-Moles, Ramos-Garcia and Warnakulasuriya 2021).

Diagnostic criteria	
Clinical criteria	- Presence of white reticular lesions at any location of the oral mucosa. The bilaterality or symmetry of the lesions is not a diagnostic criterion.
	- Presence or not of atrophic, erosive, bullous, papular or plaque lesions.
	- Exclusion criteria or criteria that make the diagnosis of OLP unlikely:
	-- Intimate contact of the lesions with dental restoration materials, essentially silveramalgam.
	-- Development of lesions in close temporal relationship with the intake of a drug.
	-- History of organ transplantation, especially bone marrow transplantation.
	-- Presence of skin lesions or systemic disorders suggestive of lupus erythematosus
Histopathologic criteria	- The histological study should seek to determine the presence and severity of epithelial dysplasia and to exclude cancer.
	- The histological study must demonstrate the characteristic facts of an autoimmune aggression - predominantly T-lymphocytic inflammatory infiltration in the band and vacuolizing degeneration and apoptosis of the basal layer of the epithelium.
	- In atypical cases direct immunofluorescence should be performed to exclude conditions such as erythematous lupus.
	Source: González-Moles, M. Á., Ramos-García, P., & Warnakulasuriya, S. (2021). An appraisal of highest quality studies reporting malignant transformation of oral lichen planus based on a systematic review. Oral diseases, 27(8), 1908-1918.
